# UV RESISTANCE LOCUS 8 signalling enhances photosynthetic resilience to herbicide‐induced damage in *Arabidopsis thaliana*

**DOI:** 10.1111/nph.70303

**Published:** 2025-06-14

**Authors:** Christopher L. Groves, Keara A. Franklin

**Affiliations:** ^1^ School of Biological Sciences University of Bristol Life Sciences Building Bristol BS8 1TQ UK

**Keywords:** Arabidopsis, *Chenopodium*, herbicide, photosynthesis, UV‐B, UVR8

## Abstract

- Perception of low irradiance ultraviolet B (UV‐B) light (280–315 nm) by the UV RESISTANCE LOCUS 8 (UVR8) photoreceptor initiates signalling pathways that enhance plant defences to UV‐B damage, mitigating the effects of higher photon irradiances. We therefore questioned whether UVR8 signalling could also prime plants against herbicide‐induced damage, promoting postspray survival.
- We assessed the effects of a 2 d, low irradiance UV‐B pretreatment on the photosynthetic resilience and survival of *Arabidopsis thaliana* plants treated with herbicides promoting photosynthetic disruption and oxidative stress.
- UV‐B acclimation increased leaf carotenoid production, antioxidant activity and nonphotochemical quenching (NPQ) and delayed herbicide‐induced reductions in electron transport rate (ETR), facilitating postspray regrowth and enhancing plant survival. In the absence of UV‐B, this protection declined within 4 d, suggesting that it is unlikely to result from structural modifications. UV‐B‐mediated enhancement of photosynthetic resilience was abolished in the *uvr8‐6* mutant and increased in the UV‐B hyper‐responsive *repressor of UV‐B photomorphogenesis1/2* (*rup1rup2*) mutant, highlighting the involvement of UVR8 signalling. UV‐B filtering during daylight acclimation also increased herbicide efficacy in *Chenopodium*, suggesting similar responses in agricultural weeds.
- UV‐B‐induced photoprotection enhances the resilience of plant photosystems to herbicide damage, providing a key target for increasing product efficacy and reducing usage.

## Introduction

Weeds significantly reduce crop yields and are a major threat to global food security (Oerke, 2006; Storkey *et al*., 2021). Perception of competing vegetation can induce shade avoidance responses in crops, prioritising stem elongation for light foraging, while reducing harvestable biomass and plant robustness (Pantazopoulou *et al*., 2021; Horvath *et al*., 2023). In addition, weeds can compete with crops for resources and release allelopathic compounds, disrupting the soil microbiome and creating significant problems for farmers (Trognitz *et al*., 2016). Agrochemical spraying is a widely adopted practice to suppress weed growth, with herbicides comprising 60% of the global pesticide market (Mohd Ghazi *et al*., 2023). The continuous evolution of resistance within weed populations, however, requires farmers to apply mixtures of herbicides with different modes of action (Diggle *et al*., 2003; Busi *et al*., 2013). Extensive herbicide use increases risks to nontarget organisms and contamination of terrestrial and aquatic ecosystems (Beketov *et al*., 2013; Mohd Ghazi *et al*., 2023; Ramos *et al*., 2023), resulting in regulatory constraints on usage and a requirement for continuous new product development.

Atrazine (6‐chloro‐N‐ethyl‐N‐(1‐methylethyl)‐1,3,5‐triazine‐2,4‐diamine; ATZ) is a triazine class herbicide commonly used in North and South America to control broadleaf and grass weeds in corn, sugar cane and sorghum. It is the most widely used Group 5 herbicide, with > 32 million kg yr^−1^ applied in the United States (Twitty & Dayan, 2024). ATZ binds to the D1 protein of photosystem II (PSII), disrupting electron flow to plastoquinone, producing triplet Chl and inducing reactive oxygen species (ROS)‐induced damage to photosynthetic machinery (Hess, 2000; Shaner, 2014). Its usage has presented multiple environmental concerns, most notably its build‐up in groundwater, as well as its suspected role as an endocrine disruptor in amphibians (Hayes *et al*., 2010). Paraquat (1,1‐dimethyl‐4,4‐bipyridylium; PQT) is a broad spectrum nonselective herbicide used in minimum‐tillage farming. It is the most widely used Group 22 herbicide, with nearly 8 million kg yr^−1^ applied in the United States (Twitty & Dayan, 2024). PQT accepts electrons at PSI, reducing molecular oxygen to form superoxide ROS, promoting significant membrane damage (Shaner, 2014). Due to its potential toxicity to nontarget organisms, PQT has been banned across 67 countries (Stuart *et al*., 2023). Understanding how environmental factors regulate plant sensitivity to herbicides may provide novel avenues to increase herbicide efficacy and reduce dosage. Light quantity, temperature, CO_2_ and humidity affect stomatal aperture and photosynthetic rate, affecting herbicide uptake and activity (Varanasi *et al*., 2016). The effect of light quality on herbicide action is, however, more poorly studied. Glasshouse experiments assaying the effects of the auxin mimic, 2,4‐D, on mustard and tomato showed an increase in herbicide efficacy in red and blue light when compared to white light controls (Datta & Dunn, 1959), whereas high‐dose ultraviolet B (UV‐B) caused a synergistic stress to aquatic fern species when applied simultaneously to the herbicide pretilachlor (Prasad *et al*., 2016). Prolonged growth of agricultural weeds in elevated UV‐B conditions simulating a 15% ozone layer depletion significantly reduced plant size and enhanced the production of cuticular waxes on leaves, reducing absorption of the herbicides PQT and glyphosate (Wang *et al*., 2007; Yin *et al*., 2012). At lower doses, UV‐B induces acclimation responses that protect plant cells from photosystem damage and oxidative stress in prolonged sunlight (Shi & Liu, 2021), so we hypothesised that short‐term UV‐B acclimation may additionally prime endogenous plant resilience to herbicides that induce ROS damage and photosynthetic disruption, such as ATZ and PQT. This idea is supported by observations that herbicides are commonly more effective in glasshouses, where UV‐B is filtered, than in the field (Clark *et al*., 2004).

Prolonged and high‐dose UV‐B can damage plant cells through the production of ROS and pyrimidine dimers in DNA, triggering the oxidation of lipids and proteins and disrupting DNA synthesis and replication (Hideg *et al*., 2013). Photosynthesis is additionally impaired through downregulation of photosynthetic proteins (Kataria *et al*., 2014). Low‐dose UV‐B is perceived by the UV RESISTANCE LOCUS 8 (UVR8) photoreceptor, a seven‐bladed β‐propeller protein dimer joined by a network of salt bridges (Rizzini *et al*., 2011; Christie *et al*., 2012). A conserved cluster of tryptophan residues at the dimer interface absorbs UV‐B, which disrupts salt bridges, initiating dimer dissociation. UVR8 monomers translocate to the nucleus, where they physically interact with the E3 ubiquitin ligase, CONSTITUTIVELY PHOTOMORPHOGENIC 1 (COP1), to initiate downstream signalling, in part through stabilisation of the bZIP transcription factors ELONGATED HYPOCOTYL 5 (HY5) and HY5 HOMOLOG (HYH) (Ulm & Jenkins, 2015; Jenkins, 2017). UVR8 signalling is attenuated through disruption of the UVR8‐COP1 interaction and redimerisation, facilitated by REPRESSOR OF UV‐B PHOTOMORPHOGENESIS (RUP) proteins, RUP1 and RUP2 (Gruber *et al*., 2010; Fang *et al*., 2022). In addition to regulating a variety of photomorphogenic responses, UVR8 signalling initiates acclimation responses that protect plant cells against the damaging effects of UV‐B radiation. These include increased production of UV‐screening compounds, antioxidant defences and DNA repair enzymes (Shi & Liu, 2021; Leonardelli *et al*., 2024). Increased production of flavonoids enhances both sunscreen and antioxidant defences and involves upregulation of the key regulatory enzyme CHALCONE SYNTHASE (CHS). Photoactivated UVR8 interacts with MYB13, which positively regulates *CHS* expression and flavonoid production (Qian *et al*., 2020). Parallel increases in ferulic acid‐5‐hydroxylase (F5H) expression additionally increase the production of UV‐B‐absorbing sinapate esters (Leonardelli *et al*., 2024). The extent of UV‐B damage is modulated by blue light, which elevates RUP levels, driving UVR8 redimerisation and activating expression of DNA repair enzymes (Tissot & Ulm, 2020; Guo *et al*., 2023). UV‐B exposure additionally increases leaf carotenoid production, which is thought to prime plants against high light stress (Badmus *et al*., 2022).

Here, we exploit genetic resources available in Arabidopsis to show that UV‐B acclimation, mediated through the UVR8/RUP signalling pathway, enhances plant tolerance to multiple widely used herbicides with modes of action involving photosynthesis disruption and ROS‐induced damage, such as ATZ and PQT. No effect of UV‐B acclimation was seen with the shikimate pathway inhibitor glyphosate (N‐phosphonomethyl glycine) and minimal effects were observed with synthetic auxins. Low‐dose UV‐B treatment (1 μmol m^−2^ s^−1^) before herbicide spraying increased *CHS* expression, carotenoid content and nonphotochemical quenching (NPQ), the process by which plants dissipate excess light energy (Müller *et al*., 2001), in mature Arabidopsis plants. UV‐B acclimation additionally decreased herbicide‐induced elevations in ROS activity and delayed herbicide‐induced reductions in photosynthetic electron transport rate (ETR), promoting regrowth and postspray survival.

## Materials and Methods

### Plant materials and growth conditions

All Arabidopsis (*Arabidopsis thaliana* (L.) Heynh.) plants used in this study were in the Columbia‐0 (Col‐0) background. *uvr8‐6* (Favory *et al*., 2009) and *rup1/2* mutants (Gruber *et al*., 2010) have been described previously. *Chenopodium amaranticolor* (also known as *Chenopodium giganteum* D.Don) seeds were obtained from the University of Bristol glasshouse collection. Seeds were sown onto a 3 : 1 mix of Levington advance F2 compost and horticultural sand before stratification in the dark at 4°C for 3 d. Arabidopsis trays were then moved to climate‐controlled growth chambers (Microclima 1600E; Snijder Scientific, Tilburg, the Netherlands) with 16 h : 8 h, light : dark cycles of white light (80 μmol m^−2^ s^−1^) at 20°C and 70% relative humidity. White light was provided by cool white fluorescent tubes (400–700 nm). UV‐B supplementation (*c*. 1.0 μmol m^−2^ s^−1^) was provided by TL T12 40W/01 G13 RS tubes (Philips Lighting, Eindhoven, the Netherlands). Negative controls for UV‐B supplementation experiments were treated with a UV‐B tube covered with 3‐mm polycarbonate to specifically block transmission of UV‐B wavelengths (Supporting Information Fig. S1a). *Chenopodium* plants were grown in natural light : dark cycles in the University of Bristol glasshouses, maintained at 65% humidity with temperature cycles of 22°C : 20°C, day : night. For daylight acclimation experiments, well‐watered 14‐d‐old plants were moved outside at 12:00 h for 3 d. Half the plants were covered in a 3‐mm polycarbonate sheet (Clear Palsun™), positioned overhead. Daylight spectra at plant height ± the polycarbonate filter are shown in Fig. S1(b). On the day of transfer, light levels at plant height were recorded as 290 μmol m^−2^ s^−1^ photosynthetically active radiation (PAR) and 1 μmol m^−2^ s^−1^ UV‐B. Underneath the filter, there was a small drop in PAR (230 μmol m^−2^ s^−1^) and significant UV‐B depletion. Weather conditions during acclimation were cloudy and dry, with temperatures ranging from 13°C to 23°C. All light measurements were recorded using a FLAME‐S‐UV‐VIS spectrometer controlled via OceanView v.2 (Ocean Optics, Osfildern, Germany). Raw text files were then analysed in Rstudio.

### Herbicide spraying

ATZ (45330; Sigma‐Aldrich) and metribuzin (36165; Merck Chemical, Darmstadt, Germany) were first dissolved in acetone (10% of final volume) before dilution to the required volume using 0.1% Genapol X‐080 (48750; Sigma‐Aldrich). PQT (36541; Sigma‐Aldrich) was dissolved in 0.1% Genapol X‐080. Mesotrione (Callisto® and Calaris®), glyphosate (Touchdown Total®), Perlargonic acid (Roundup® NL Glyphosate Free) and Fluroxypyr/Clopyralid/MCPA (Weedol®) were obtained as commercial products and dissolved in reverse osmosis (RO) water. Plants were sprayed using a custom‐designed track sprayer fitted with an 80 02 EVS nozzle (TeeJet Technologies, Wisbech, UK). Herbicide spray volume was calculated before each experiment using preweighed Petri dishes containing three filter papers sprayed 10 times with water. The dishes were then re‐weighed to give the deposit in grams. The amount applied over the Petri dish from 10 runs was converted into spray volume (l ha^−1^) using the equation below, assuming 1 g = 1 ml water (adapted from Laycock, 2004):

$$\text{Application volume}\;\left(l\;{\mathrm{ha}}^{-1}\right)=\frac{\text{Deposit after}\;10\;\text{runs}\;\left(g\right)\times 10\;000}{\text{Area of petri dish}\;\left({\mathrm{cm}}^{2}\right)}$$

Herbicide treatments were quantified as grams of active ingredient (gai) per hectare (ha). The amount of product required for formulation was calculated using the equation below (adapted from Laycock, 2004):

$$\begin{array}{l} \text{Amount of herbicide required}\;\left(\mu l\;\text{or}\;\mathrm{mg}\right)\\ \;=\frac{\text{Rate required}\;\left(\mathrm{gai}\;{\mathrm{ha}}^{-1}\right)\times \text{Total volume}\;\left(\mathrm{ml}\right)\times \frac{100}{\%\mathrm{ai}\;\text{in working stock}}}{\text{Application volume}\;\left(l\;{\mathrm{ha}}^{-1}\right)} \end{array}$$

All herbicide spraying was performed at midday to control for circadian gating in herbicide sensitivity and/or activity (Belbin *et al*., 2019). Plant survival was defined by the presence of an active shoot apical meristem and newly developed green tissue visible 14 d after herbicide application.

### Chlorophyll and carotenoid quantification

Plant tissue samples were harvested from rosette leaf 2, weighed and snap frozen in liquid nitrogen. Tissue was homogenised using a TissueLyser II (Qiagen) for 4 min at 30 Hz, and 1.5 ml of ice cold 80% acetone was added to each sample. Samples were then incubated in the dark at 4°C for 24 h. These were centrifuged at 4°C for 10 min at 30 000 ***g***, and the supernatant was diluted to 2 ml with 80% acetone. Absorbances were recorded at 646, 663 and 470 nm, and Chl/carotenoid contents were calculated as described in Wellburn (1994).

### RT‐qPCR

Fifty milligram samples of rosette leaf 2 tissue were snap frozen in liquid nitrogen and homogenised using a Qiagen TissueLyser II for 2 min at 30 Hz. RNA was extracted using Spectrum™ Plant Total RNA Kit (Merck). One microgram of RNA from each sample was used for cDNA synthesis using a High Capacity cDNA Reverse Transcription Kit (Applied Biosystems, Waltham, MA, USA). Reverse transcription quantitative polymerase chain reaction (RT‐qPCR) was performed using Brilliant III Ultra‐Fast SYBR® Green QPCR Master Mix and ROX as the reference dye. Relative transcript abundance was calculated using the $2^{-\Delta \Delta C_{T}}$ method (Livak & Schmittgen, 2001) and normalised to *ACTIN2*. Primer sequences are provided in Table S1.

### Chlorophyll fluorescence

Chl fluorescence was measured using the MAXI IMAGING‐PAM M‐Series system, running ImagingWin v.2.56 (Walz, Effeltrich, Germany). Plants were dark‐adapted for 20 min before measurement. Excitation light was provided at 450 nm, and the saturating pulse was set to intensity 2. Rapid light curves (RLC) were performed using custom programs described in the figure legend of each experiment. Induction curves were performed using ImagingWin. Photosynthetic parameters were calculated as described in Baker (2008), Maxwell & Johnson (2000) and Murchie & Lawson (2013). These are detailed in Table 1.

**Table 1:** Measured and calculated Chl fluorescence parameters.

Parameter	Definition	Equation
*F*_0_	Minimal fluorescence (dark‐adapted)	
*F*_m_	Maximum fluorescence (dark‐adapted)	
*F*′	Minimal fluorescence (light‐adapted)	
*F*_m_′	Maximum fluorescence (light‐adapted)	
*F*_v_/*F*_m_	Maximum quantum yield of photosystem II (PSII) (dark‐adapted)	(*F*_m_ − *F*_0_)/*F*_m_
Y(II)/^Φ^PSII	Quantum yield of PSII	(*F*_m_′ − *F*′)/*F*_m_′
NPQ	Nonphotochemical quenching	(*F*_m_ − *F*_m_′)/*F*_m_′
ETR	Electron transport rate	Y(II) × PAR × 0.5 × 0.84

### ROS quantification

ROS were quantified using the fluorescent probe 2′,7′‐dichlorodihydrofluorescein diacetate (H2DCFDA). This was dissolved in dimethyl sulfoxide (DMSO), and a 25 μM stock was prepared in 50 mM phosphate buffer at pH 7.4. The first rosette leaves were excised from 23‐d‐old plants and secured to Petri dish lids using tape. These were covered in buffer and incubated in white light at 20°C for 60 min to allow recovery. Leaves were then transferred to 25 μM H2DCFDA for 20 min, washed in RO water and imaged to record pretreatment ROS levels. For herbicide treatment, leaves were submerged in 50 mM phosphate buffer (pH 7.4) containing 0.423 mM PQT and 0.1% DMSO, or formulation controls. They were then washed with RO water, and whole leaves were imaged using a Leica MZ FL III fluorescence microscope (Leica Microsystems, Wetzlar, Germany) and DP74 CMOS 20.7 megapixel cooled colour camera (Olympus, Tokyo, Japan), controlled by the CellSens Standard v.2 advanced imaging software. Illumination was provided by pE‐300^white^ LEDs (CoolLED) and images were exported to TIFF files, which were analysed in Fiji.

### Data analysis

Data were analysed using Rstudio, plotted using the ggplot2 package from tidyverse, and processed in Inkscape.

## Results

### UV‐B acclimation enhances photosystem resilience and plant survival following ATZ treatment

**Fig. 1:**
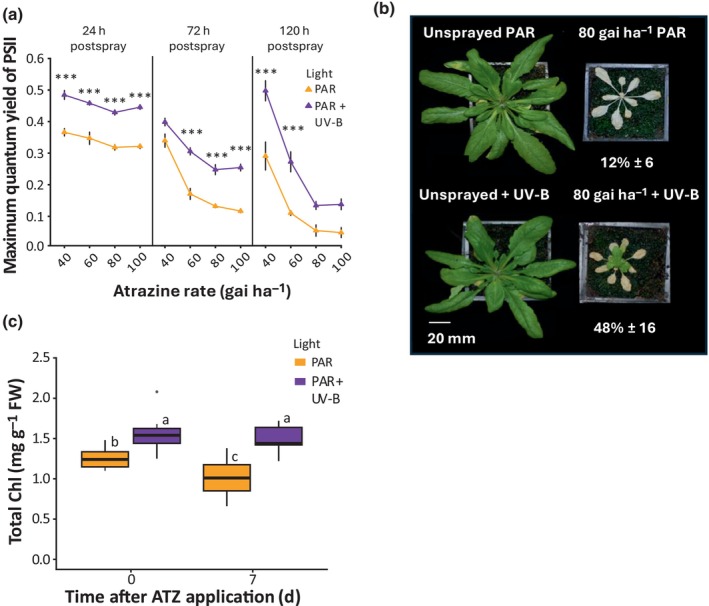
Low‐dose ultraviolet B (UV‐B) treatment enhances Atrazine (ATZ) tolerance. (a) *F*_v_/*F*_m_ of Arabidopsis plants following UV‐B supplementation and ATZ treatment at different rates. Plants were grown on soil for 21 d in white light (photosynthetically active radiation (PAR): 80 μmol m^−2^ s^−1^) at 20°C. Half the plants were then treated with supplementary narrowband UV‐B at 1 μmol m^−2^ s^−1^ for 2 d before ATZ treatment (PAR + UV‐B). Four rates of ATZ were sprayed (40, 60, 80 and 100 gai ha^−1^) in a 10% acetone + 0.1% Genapol X‐080 formulation at 2 h postdawn. Data points represent mean values, and bars represent SEM. *n* = 10. Differences between light treatments at each rate were calculated using a two‐way ANOVA and Tukey's *post hoc* test. ***, *P* < 0.001. (b) The effect of UV‐B acclimation on plant survival following ATZ treatment. Plants were grown and treated as in (a). Survival (represented as %) was determined by the presence of green tissue 14 d after spraying. *n* = 72 over eight experiments (PAR) and 42 over five experiments (PAR + UV‐B). Variation is shown as SEM between % survival values (c) The effect of 80 gai ha^−1^ ATZ treatment on leaf Chl content, with and without UV‐B‐acclimation. Plants were grown and treated as in (a). Total Chl was extracted from rosette leaf 2 at 0 and 7 d postspray. Data are presented as boxplots showing the median and interquartile range of each group. The upper and lower whiskers represent data within 1.5 × IQR. Dots represent outliers. Different letters represent significant differences (*P* < 0.05). *n* = 12.

To investigate whether UV‐B acclimation affects plant herbicide tolerance, Arabidopsis plants were grown on soil in 80 μmol m^−2^ s^−1^ of PAR for 21 d. UV‐B supplementation (280–315 nm; 1 μmol m^−2^ s^−1^) was provided at dawn on Day 21 for 48 h. 1 μmol m^−2^ s^−1^ is sufficient to initiate UV‐B‐induced photomorphogenic responses, without the stress‐induced damage observed at higher photon irradiances (Hayes *et al*., 2014). UV‐B supplementation was removed before herbicide spraying to ensure that any observed effects resulted from plant acclimation and not UV‐B effects on herbicide stability. ATZ was selected as a fast‐acting herbicide targeting PSII. Plants were sprayed at midday on Day 23 with a range of application rates (40, 60, 80 and 100 gai ha^−1^), selected to display incremental damage in Arabidopsis. Photosystem performance was monitored through measurements of the maximum quantum yield of PSII (variable fluorescence/maximum fluorescence; *F*_v_/*F*_m_) at 24, 72 and 120 h postspray (Maxwell & Johnson, 2000). In white light (PAR) controls, ATZ application reduced *F*_v_/*F*_m_ from 0.82 to 0.37 (40 gai ha^−1^), 0.35 (60 gai ha^−1^) and 0.32 (80 and 100 gai ha^−1^) within 24 h, confirming herbicide uptake and activity (Fig. 1). Rate‐dependent reductions in *F*_v_/*F*_m_ were observed at all time points. UV‐B acclimated plants (PAR + UV‐B) displayed higher *F*_v_/*F*_m_ values than controls (PAR) in all treatments, suggesting greater photosystem performance in these plants (Fig. 1a). To assess whether UV‐B‐induced changes in *F*_v_/*F*_m_ impact herbicide efficacy, plant survival was rate scored 14 d after ATZ spraying at 80 gai ha^−1^. In this experiment, UV‐B acclimation enhanced plant survival from 12% to 48%, with visible leaf regrowth observed in surviving plants (Fig. 1b). The Chl content of rosette leaf 2 was significantly increased in UV‐B acclimated plants at 7 d postspray, consistent with delayed tissue death (Fig. 1c). To assess the longevity of UV‐B acclimation, the length of time between UV‐B acclimation and ATZ spraying was gradually extended and *F*_v_/*F*_m_ assessed 24 h after application. Plants were grown as described in Fig. 1(a), but a lower rate of ATZ (20 gai ha^−1^) used to slow the rate of *F*_v_/*F*_m_ decline. As the length of time between UV‐B treatment and ATZ application increased, the effect of UV‐B acclimation diminished, with no significant effect observed after 4 d (Fig. S2). It can therefore be concluded that the enhancement of photosystem performance induced by UV‐B acclimation is transient, with UV‐B exposure immediately before spraying showing greatest effectiveness.

### UV‐B acclimation delays ATZ‐induced reductions in electron transport rate

**Fig. 2:**
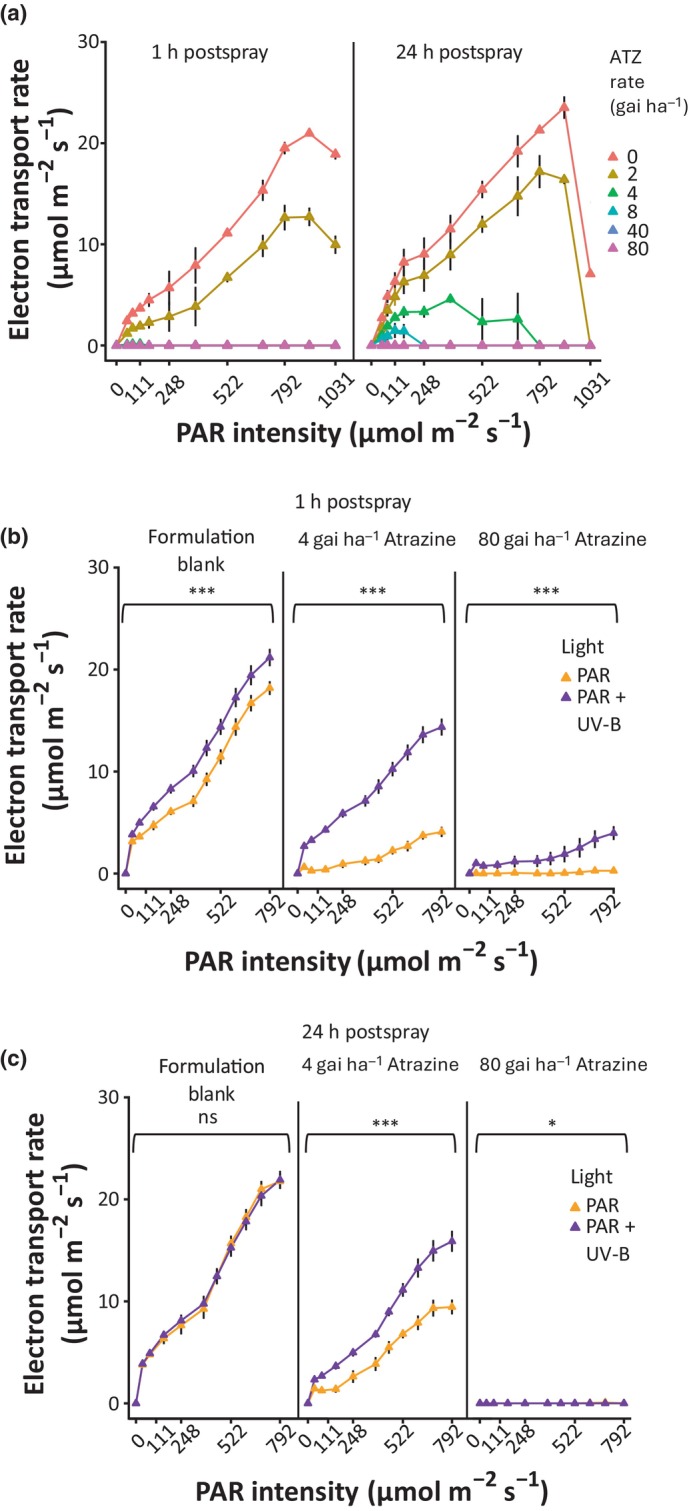
Low‐dose ultraviolet B (UV‐B) treatment delays Atrazine (ATZ)‐induced reductions in electron transport rate (ETR). (a) ETR of Arabidopsis plants following UV‐B supplementation and ATZ treatment at different rates. Plants were grown on soil for 21 d in white light (photosynthetically active radiation (PAR): 80 μmol m^−2^ s^−1^) at 20°C. ATZ was sprayed in a 10% acetone 0.1% Genapol X‐080 formulation at 0, 2, 4, 8, 40 and 80 gai ha^−1^ at 2 h postdawn. ETR was measured 1 and 24 h later using a rapid light curve (RLC), with saturating pulses every 30 s and actinic light applied in 0, 0, 50, 76, 111, 153, 248, 372, 522, 689, 792, 908, 1031 μmol m^−2^ s^−1^ steps. (b, c) Effect of UV‐B acclimation on ETR following ATZ application. Plants were grown as in (a) but half were treated with supplementary narrowband UV‐B at 1 μmol m^−2^ s^−1^ for 2 d before ATZ treatment (PAR + UV‐B). ATZ was sprayed at 4 and 80 gai ha^−1^. ETR was measured 1 h (b) and 24 h (c) later using a RLC, with saturating pulses every 20 s and actinic light applied in 0, 36, 76, 153, 248, 372, 445, 522, 605, 698 and 792 μmol m^−2^ s^−1^ steps. *n* = 3 (a) and 7–8 (b, c). Data points represent mean values, and bars represent SEM. Differences between light treatments at each rate were calculated using a two‐way ANOVA and Tukey's *post hoc* test. *, *P* < 0.05; **, *P* < 0.01; ***, *P* < 0.001.

To gain further insight into the impact of UV‐B acclimation on photosynthetic function, ETR were calculated following ATZ application. This was achieved by measuring the quantum yield of photosynthesis (Y(II)) at increasing light intensities (Maxwell & Johnson, 2000). ETR declined rapidly within 1 h of herbicide application at ATZ rates > 2 gai ha^−1^ (Fig. 2a). By 24 h, ETR had recovered in plants sprayed at 4 gai ha^−1^ but remained suppressed in plants sprayed with higher rates. The impact of UV‐B acclimation on ETR was assessed following ATZ spraying at both 4 and 80 gai ha^−1^. A small UV‐B‐mediated increase in ETR was observed in formulation controls at 1 h but was not visible at 24 h postspray (Fig. 2b,c). At 4 gai ha^−1^, UV‐B‐treated plants showed significantly higher ETRs than controls at both 1 and 24 h postspray. At 80 gai ha^−1^ ATZ, UV‐B effects were only visible 1 h postspray (Fig. 2b,c). Collectively, these data show that UV‐B acclimation can delay ATZ‐induced reductions in ETR, potentially facilitating regrowth and survival (Fig. 1b).

### UV‐B acclimation increases carotenoid production and nonphotochemical quenching

**Fig. 3:**
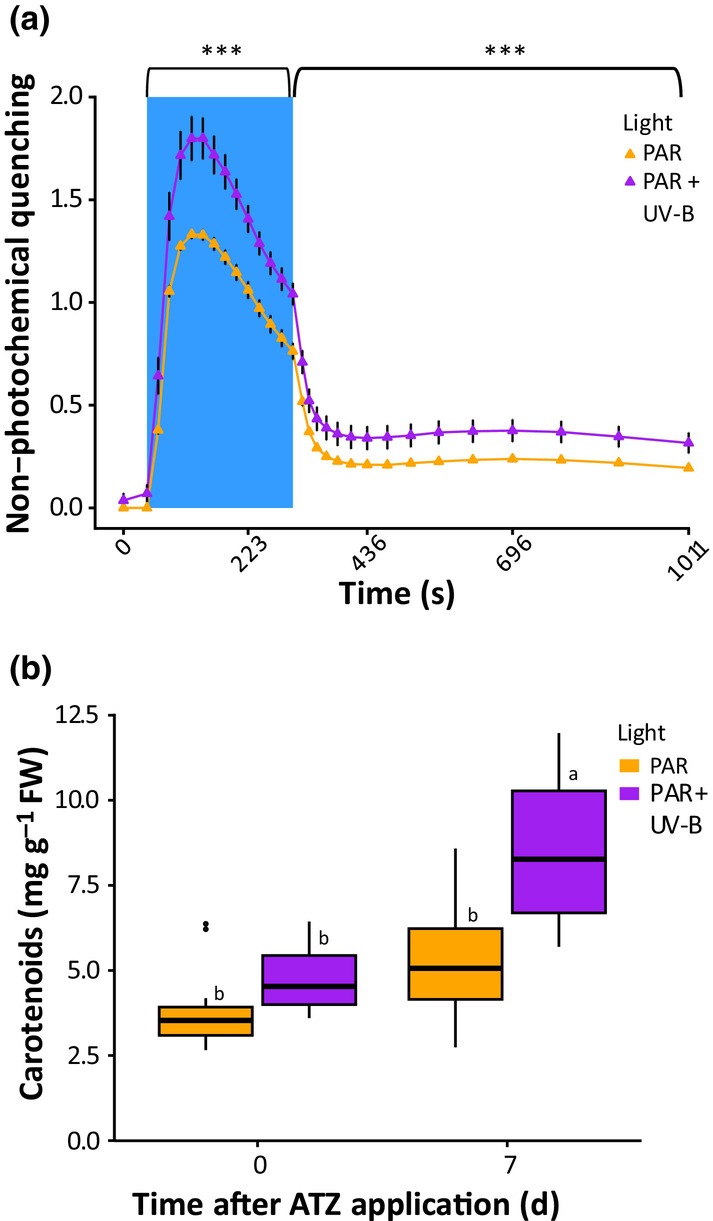
Low‐dose ultraviolet B (UV‐B) increases carotenoid production and enhances nonphotochemical quenching (NPQ). (a) The effect of UV‐B acclimation on NPQ. Arabidopsis plants were grown on soil for 21 d in white light (photosynthetically active radiation (PAR): 80 μmol m^−2^ s^−1^) at 20°C. Half the plants were then treated with supplementary narrowband UV‐B at 1 μmol m^−2^ s^−1^ for 2 d. NPQ was measured at 2 h postdawn using an induction curve followed by recovery. The induction curve was performed by making an initial *F*_v_/*F*_m_ measurement and then Y(II) measurement in 76 μmol m^−2^ s^−1^ actinic light every 20 s for 5 min (blue background). Actinic light was then switched off, and Y(II) measured over the next 10 min at increasingly longer intervals to measure recovery. *n* = 5. Data points represent mean values, and bars represent SEM. Differences between light treatments were calculated using a two‐way ANOVA. ***, *P* < 0.001. (b) The effect of ATZ treatment on leaf carotenoid content, with and without UV‐B acclimation. Plants were grown and treated as in (a) before spraying with Atrazine (ATZ) at 80 gai ha^−1^ at 2 h postdawn. Total carotenoids were extracted from rosette leaf 2 at 0 and 7 d postspray. Data are presented as boxplots showing the median and interquartile range of each group. The upper and lower whiskers represent data within 1.5 × IQR. Dots represent outliers. Different letters represent significant differences (*P* < 0.05). *n* = 12.

High light can induce the production of excited singlet and triplet state Chl, driving photooxidative stress in chloroplasts. These effects are mitigated through dissipation of excess energy as heat by the process of NPQ. Excess light reduces thylakoid lumen pH, initiating conformational changes within light harvesting complexes and de‐epoxidation of the carotenoid violaxanthin to zeaxanthin, termed the xanthophyll cycle (Müller *et al*., 2001). High‐dose UV‐B has been shown to reduce NPQ in detached leaves of tomato, cucumber and Arabidopsis (Moon *et al*., 2011). Conversely, lower levels of UV‐B perceived via the UVR8 photoreceptor have been shown to enhance violaxanthin production and NPQ in *Chlamydomonas reinhardtii*, reducing photodamage (Allorent *et al*., 2016). We therefore questioned whether UV‐B acclimation would affect carotenoid content and NPQ capacity in Arabidopsis leaves. Plants which received 2 d of UV‐B supplementation displayed higher NPQ values and leaf carotenoid content than white light controls (Fig. 3a,b). As ATZ induces leaf damage through triplet Chl production, similarly to high light (Hess, 2000; Shaner, 2014), enhanced carotenoid quenching may contribute towards the increased ATZ tolerance observed in UV‐B acclimated plants.

### UV‐B enhancement of ATZ tolerance is mediated by UVR8 signalling

**Fig. 4:**
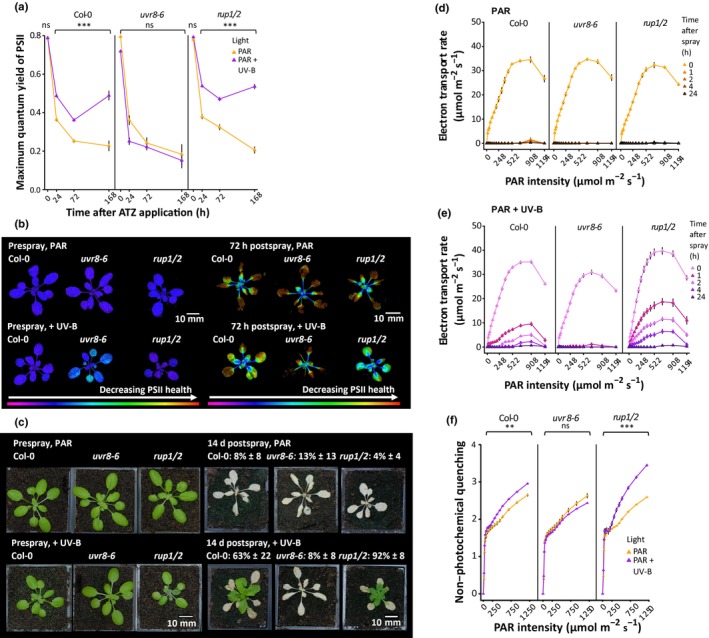
Ultraviolet B (UV‐B)‐mediated promotion of Atrazine (ATZ) tolerance involves UV RESISTANCE LOCUS 8 (UVR8) signalling. (a) The role of UVR8 signalling in UV‐B‐mediated *F*_v_/*F*_m_ enhancement following ATZ application. Arabidopsis Columbia‐0 (Col‐0), *uvr8‐6* and *rup1/2* plants were grown on soil for 21 d in white light (PAR: 80 μmol m^−2^ s^−1^) at 20°C. Half the plants were then treated with supplementary narrowband UV‐B at 1 μmol m^−2^ s^−1^ for 2 d before ATZ spraying (PAR + UV‐B). ATZ was sprayed at 80 gai ha^−1^ in a 10% acetone + 0.1% Genapol X‐080 formulation at 2 h post‐dawn. Data points represent mean *F*_v_/*F*_m_ values, and bars represent SEM. *n* = 10. Differences between light treatments in each genotype were calculated using a two‐way ANOVA and Tukey's *post hoc* test. ***, *P* < 0.001. (b) Chl fluorescence images from before and 72 h after ATZ application. Photosystem II (PSII) health is displayed as a false colour image, with purple representing a *F*_v_/*F*_m_ value of 1, and black, 0 (complete breakdown of PSII). A healthy plant will have a *F*_v_/*F*_m_ value of *c*. 0.8 (dark blue). (c) The role of UVR8 signalling in UV‐B‐mediated ATZ tolerance. Col‐0, *uvr8‐6* and *rup1/2* plants were grown and treated as in (a). Survival (represented as %) was determined by the presence of green tissue 14 d after spraying. For both PAR and +UV‐B, *n* = 24 across three experiments. Variation is shown as SE between % survival values. (d, e) The role of UVR8 signalling on UV‐B‐mediated ETR enhancement following ATZ application. Col‐0, *uvr8‐6* and *rup1/2* plants were grown and treated as in (a). ETR values were recorded before and after ATZ treatment for PAR (d) and PAR + UV‐B (e) treated plants. Plants were illuminated with 0, 20, 36, 50, 76, 111, 153, 198, 248, 308, 372, 445, 522, 698, 908 and 1194 μmol m^−2^ s^−1^ actinic light in 30 s steps, and Y(II) measured at the end of each step, which was then used to calculate ETR. *n* = 4 (nonacclimated) and 10 (UV‐B acclimated). Data points represent mean values, and bars represent SEM. (f) The role of UVR8 signalling in UV‐B‐mediated NPQ enhancement. Col‐0, *uvr8‐6* and *rup1/2* plants were grown and treated as in (a). Plants were illuminated with 0, 20, 36, 50, 76, 111, 153, 198, 248, 308, 372, 445, 522, 698, 908 and 1194 μmol m^−2^ s^−1^ actinic light in 30 s steps, and NPQ measured at the end of each step. *n* = 4 (nonacclimated plants) and 10 (UV‐B acclimated plants). Data points show mean values, and bars represent SEM. For each genotype, differences between light treatments were identified using two‐way ANOVA testing for the interaction between light treatment and PAR intensity; **, *P* < 0.01; ***, *P* < 0.001; ns, nonsignificant.

To assess the contribution of UVR8 signalling to UV‐B‐mediated herbicide tolerance, *uvr8‐6* mutants lacking the UVR8 photoreceptor (Favory *et al*., 2009) and UV‐B hyper‐responsive *rup1/2* mutants, deficient in UVR8 redimerisation (Gruber *et al*., 2010) were analysed alongside wild‐type (WT) controls, following the same protocols as described in Figs 1, 2, 3. Twenty‐one‐day‐old plants were treated with 2 d of white light ± UV‐B to induce UV‐B acclimation before ATZ spraying at 80 gai ha^−1^. Maximum quantum yield of PSII (*F*_v_/*F*_m_), ETR and NPQ were all measured in separate experiments, with % survival recorded for each (Fig. 4a–f).

Before spraying, UV‐B acclimated and unacclimated WT plants displayed similar *F*_v_/*F*_m_ and ETR values (Fig. 4a,b,e). *uvr8‐6* plants displayed only small decreases in *F*_v_/*F*_m_ and ETR, confirming that the low irradiance of UV‐B used for acclimation treatment had not initiated major stress responses in these plants (Fig. 4a–e). *F*_v_/*F*_m_ measurements were significantly higher in UV‐B‐treated WT and *rup1/2* plants than nonacclimated controls at 24, 72 and 168 h following ATZ treatment. *uvr8‐6* mutants resembled nonacclimated controls, suggesting a role for UVR8 signalling in maintaining photosystem performance. Consistent with data shown in Fig. 1(a), UV‐B acclimation increased the survival of WT plants post‐ATZ spraying from 8% to 63%. A slight decrease in survival rate was recorded in *uvr8‐6* mutants (13–8%), which may represent the mild stress observed in these plants before ATZ application (Fig. 4a,b). Considerably enhanced survival rates (4–92%) were observed in *rup1/2* mutants, consistent with the enhanced UVR8 signalling in these plants (Gruber *et al*., 2010). In accordance with data shown in Fig. 2(b), UV‐B acclimation increased ETR in WT plants for at least 4 h post‐ATZ treatment (Fig. 4d,e). This response was absent in *uvr8‐6* mutants and enhanced in *rup1/2* mutants, suggesting a role for UVR8 signalling. A similar trend was observed in NPQ measurements (Fig. 4f). Collectively, these data suggest that the enhanced photosynthetic resilience and survival rates in UV‐B acclimated plants following ATZ application require UVR8 signalling.

CHS is the first committed enzyme in the flavonoid biosynthetic pathway and catalyses the conversion of 4‐coumaroyl CoA to naringenin chalcone (Li *et al*., 1993). Transcript levels increase rapidly following UV‐B perception in a UVR8‐dependent manner, so they are often used as a molecular marker in UV‐B signalling studies (Brown *et al*., 2005). Increased *CHS* transcript was observed in WT leaves following UV‐B acclimation (Fig. S3). This response was sustained for 1 h post‐ATZ spray (80 gai ha^−1^) and was absent in *uvr8‐6* mutants. Significantly enhanced elevations in *CHS* transcript were observed in UV‐B acclimated *rup1/2* mutants at 1, 2 and 4 h postspray (Fig. S3). Flavonoids possess high antioxidant capacity (Dias *et al*., 2021), so increased production during UV‐B acclimation, facilitated by elevated *CHS* transcript, may contribute towards ATZ protection in WT and *rup1/2* plants.

The effect of UV‐B acclimation on four other gene transcripts with potential roles in ATZ tolerance was investigated alongside *CHS*. *PsbA* encodes the D1 protein of PSII, the target of ATZ. Increased *PsbA* gene expression has been observed in ATZ‐resistant *Commelina communis*, conferring target site resistance (Yang *et al*., 2022). UV‐B acclimation did not affect *PsbA* transcript abundance in WT plants before or following ATZ spraying (Fig. S3). Reduced *PsbA* transcript abundance was, however, observed in UV‐B‐treated *uvr8‐6* mutants before spraying, suggesting that UVR8 signalling may maintain *PsbA* transcript levels in these conditions. Consistent with this hypothesis, increased *PsbA* transcript abundance was recorded in UV‐B acclimated *rup1/2* mutants pre and post‐ATZ spraying when compared to nonacclimated controls (Fig. S3). Glutathione‐S‐transferases (GSTs) are enzymes that conjugate glutathione to a range of electrophilic molecules and perform a central role in the detoxification of xenobiotics. Sucrose‐mediated ATZ tolerance has been suggested to involve *GSTF8* induction (Sulmon *et al*., 2006). We therefore questioned whether UV‐B‐mediated acclimation also involves *GSTF8*. Consistent with published findings (Sulmon *et al*., 2006), *GSTF8* transcript levels increased following ATZ application, but no effect of UV‐B acclimation was observed (Fig. S3). Ascorbate peroxidase (APX) and catalase (CAT) enzymes perform important roles in scavenging and detoxifying ROS (Szechyńska‐Hebda *et al*., 2022), with APX activity increasing following UV‐B exposure (Rao *et al*., 1996). CAT3 performs a significant role during abiotic stress (Szechyńska‐Hebda *et al*., 2022). In Arabidopsis, both *APX6* and *CAT3* show the highest expression in mature leaves, so they were selected for analysis following UV‐B and ATZ treatment (Szechyńska‐Hebda *et al*., 2022). No consistent effect of UV‐B was observed for *APX6*, but *CAT3* displayed ATZ‐induced induction in a response requiring UV‐B and UVR8 signalling (Fig. S3). Collectively, these data suggest that UV‐B acclimation mediated by UVR8 may contribute to ATZ tolerance, in part, through enhancing CAT3 abundance and ROS detoxification following ATZ treatment.

### UV‐B acclimation enhances plant tolerance to multiple herbicides involving photosynthetic inhibition and oxidative stress

**Fig. 5:**
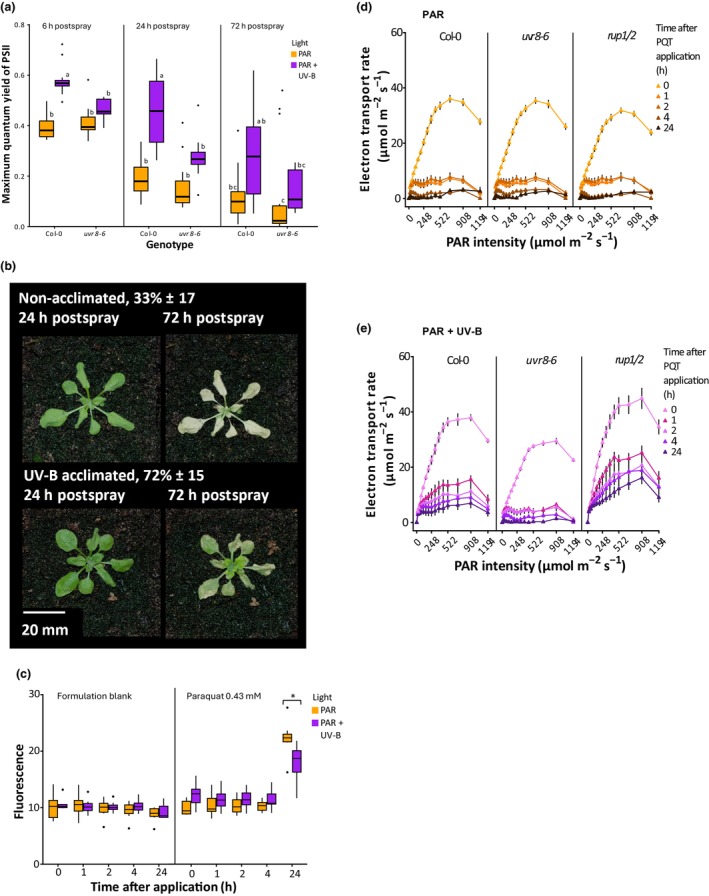
UV RESISTANCE LOCUS 8 (UVR8) signalling promotes Paraquat (PQT) tolerance. (a) *F*_v_/*F*_m_ of Arabidopsis Columbia‐0 (Col‐0) and *uvr8‐6* plants treated with and without ultraviolet B (UV‐B) acclimation, following PQT spraying. Plants were grown on soil for 14 d in white light (photosynthetically active radiation (PAR): 80 μmol m^−2^ s^−1^) at 20°C. Half the plants were then treated with supplementary narrowband UV‐B at 1 μmol m^−2^ s^−1^ for 2 d before PQT spraying (PAR + UV‐B). PQT was applied at 10 gai ha^−1^ in a 0.1% Genapol X‐080 formulation 2 h postdawn. *F*_v_/*F*_m_ values were recorded at 6, 24 and 72 h postspray. A two‐way ANOVA comparing the interaction between light and genotype with a Tukey's *post hoc* test was used to identify differences between treatments. Data are presented as boxplots showing the median and interquartile range of each group. The upper and lower whiskers represent data within 1.5 × IQR. Dots represent outliers. Different letters represent significant differences (*P* < 0.05). *n* = 12. (b) The effect of UV‐B acclimation on plant survival following PQT treatment. Plants were grown and treated as in (a) before spraying with PQT at 20 gai ha^−1^. Survival (represented as %) was determined by the presence of green tissue 14 d after spraying. For both PAR and +UV‐B, *n* = 12 across three experiments. Variation is shown as SEM between % survival values. (c) The effect of UV‐B acclimation on reactive oxygen species (ROS) production following PQT treatment. Plants were grown for 21 d as in (a) and half treated with supplementary narrowband UV‐B at 1 μmol m^−2^ s^−1^ for 2 d before PQT spraying. ROS production was measured using the cell permeable fluorescent stain H2DCFDA. First rosette leaves were excised and submerged in 50 mM pH 7.4 phosphate buffer containing 25 μM H2DCFDA for 20 min to obtain a baseline fluorescence reading before herbicide treatment. Leaves were then submerged in either a blank formulation (0.1% DMSO in 50 mM pH 7.4 phosphate buffer) or 0.43 mM PQT, and fluorescence recorded after 1, 2, 4 and 24 h. A two‐way ANOVA with a Tukey's *post hoc* test was used to identify differences between treatments. Data are presented as boxplots showing the median and interquartile range of each group. The upper and lower whiskers represent data within 1.5 × IQR. Dots represent outliers. Different letters represent significant differences (*, *P* < 0.05). *n* = 7. (d, e) The role of UVR8 signalling in UV‐B‐mediated ETR enhancement following PQT application. Col‐0, *uvr8‐6* and *rup1/2* plants were grown for 21 d as in (a) and half treated with supplementary narrowband UV‐B at 1 μmol m^−2^ s^−1^ for 2 d before PQT spraying at 20 gai ha^−1^. ETR values were recorded before and after PQT treatment in PAR (d) and PAR + UV‐B (e) treated plants. Plants were illuminated with 0, 20, 36, 50, 76, 111, 153, 198, 248, 308, 372, 445, 522, 698, 908 and 1194 μmol m^−2^ s^−1^ actinic light in 30 s steps, and Y(II) measured at the end of each step, which was then used to calculate ETR. Data points represent mean values, and bars represent SEM. *n* = 6.

To investigate the role of UV‐B acclimation in herbicide tolerance beyond ATZ, similar experiments were performed with the potent ROS inducer, PQT. Fourteen‐day‐old plants were UV‐B acclimated as described previously and sprayed with PQT at 10 gai ha^−1^. Significant reductions in *F*_v_/*F*_m_ (0.78–0.40) were observed in WT plants at 6 h postspray, which declined further at 24 and 72 h (Fig. 5a). UV‐B acclimation delayed this decline in a UVR8‐dependent manner and enhanced postspray survival from 33% to 72% (Fig. 5a,b). As PQT functions through the production of superoxide radicals (Shaner, 2014), we quantified ROS levels in detached leaves incubated in 0.43 mM PQT for up to 24 h. This concentration was calculated to equate to 10 gai ha^−1^. H_2_DCFDA is a cell permeable dye that oxidises to form dichlorodihydrofluorescein (DCF), which fluoresces when excited at 495 nm, emitting at 520 nm (Oparka *et al*., 2016). Elevated fluorescence was observed from leaves incubated in PQT, but not formulation controls (Fig. 5c). Significantly less fluorescence was recorded in UV‐B acclimated leaves than WT controls, following PQT incubation. These observations suggest that UV‐B may enhance leaf antioxidant capacity (Fig. 5c). PQT application also reduced ETR within 1 h of spraying. This decline was delayed following UV‐B acclimation in a UVR8‐dependent manner (Fig. 5d,e). *rup1/2* mutants displayed higher ETR at all timepoints, supporting a role for UVR8 signalling in promoting photosynthetic resilience (Fig. 5d,e).

The role of UVR8 signalling in enhancing photosynthetic resilience to herbicides was additionally tested with Mesotrione, Metribuzin, Glyphosate (Touchdown Total®), Perlargonic Acid (Roundup® NL Glyphosate Free) and Fluroxypyr + Clopyralid +MCPA (Weedol lawn®) applied at two different application rates. Mesotrione (2‐[4‐(methysulfonyl)‐2‐nitrobenzoyl]‐1,3‐cyclohexanedione) inhibits carotenoid synthesis while Metribuzin (4‐amino‐6‐(1,1‐dimethylethyl)‐3‐(methylthio)‐1,2,4‐triazin‐5(4H)‐one) inhibits PSII. Both are used for pre‐ and postemergence control of broadleaf weeds. Glyphosate (N‐phosphonomethyl glycine) inhibits enolpyruvyl shikimate‐3‐phosphate (EPSP) synthase activity and the biosynthesis of aromatic amino acids. Perlargonic Acid (CH_3_(CH_2_)_7_CO_2_H, *n*‐nonanoic acid) disrupts cellular membranes, driving ROS production, while Fluoxypyr ([(4‐amino‐3,5‐dichloro‐6‐fluoro‐pyridinyl)oxy]acetic acid), Clopyralid (3,6‐dichloro‐2‐pyridinecarboxylic acid) and MCPA ((4‐chloro‐2‐methylphenoxy) acetic acid) are auxin mimics. All are used for postemergence control of broadleaf weeds (Shaner, 2014; Ciriminna *et al*., 2019). UV‐B acclimated plants displayed enhanced *F*_v_/*F*_m_ values following spraying with Mesotrione and Metribuzin at both concentrations in a UVR8‐dependent manner. A similar result was observed for Perlargonic Acid at the lower concentration. UVR8‐mediated reductions in herbicide efficacy were also observed with Fluoxypyr/Clopyralid/MCPA, but these effects were smaller than with the other herbicides tested. Conversely, no effect of UV‐B acclimation was observed on Glyphosate tolerance (Fig. S4).

### UV‐B filtering enhances herbicide efficacy in daylight‐acclimated *Chenopodium* plants

To apply our findings to a more realistic agricultural scenario, we assessed the impact of UV‐B filtering on the herbicide tolerance of glasshouse‐grown *C. amaranticolor* (also known as *C. giganteum*), a fast‐growing invasive species and relative of the problematic agricultural weed *Chenopodium album* (Tang *et al*., 2022). Plants were grown under natural lighting in a glasshouse, before daylight acclimation outside for 3 d. Half the plants were covered with an overhead polycarbonate sheet to filter UV‐B (Fig. S1b). Following daylight acclimation ± UV‐B filtering, plants were sprayed with four rates (12.5, 25, 50 and 100 gai ha^−1^) of a commercial mesotrione‐based herbicide commonly used for *Chenopodium* control (Calaris®), before being returned to the glasshouse to assess herbicide efficacy. Measurements performed before herbicide spraying showed a slight *F*_v_/*F*_m_ reduction in plants acclimated to daylight without the UV‐B filter (0.69) when compared to those with (0.72). Following return to the glasshouse for 24 h postspraying, *F*_v_/*F*_m_ values for both sets of plants converged, before plants acclimated under the UV‐B filter showed a steeper decline at all herbicide rates (Fig. S5a). Daylight acclimation also increased plant survival rates, with the most notable differences observed at 50 gai ha^−1^ (Fig. S5b). Although plants situated under the polycarbonate filter may have additionally experienced small differences in PAR, humidity and airflow during UV‐B filtering, these effects did not adversely affect *F*_v_/*F*_m_. Data from this experiment support climate‐controlled Arabidopsis experiments suggesting that UV‐B acclimation reduces herbicide efficacy.

## Discussion

**Fig. 6:**
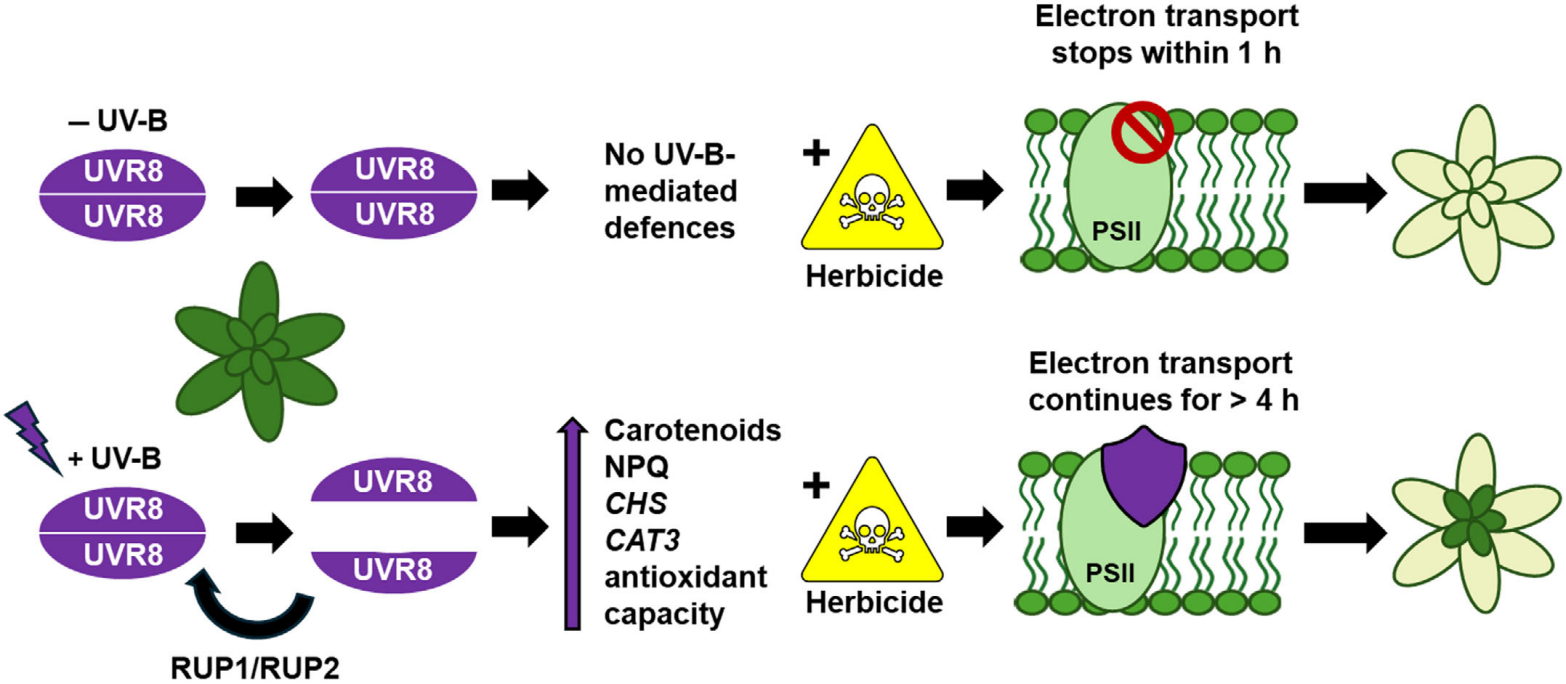
Hypothetical model of how UV RESISTANCE LOCUS 8 (UVR8) signalling promotes tolerance to herbicides with modes of action involving photosystem II (PSII) disruption and reactive oxygen species (ROS) generation. In the absence of ultraviolet B (UV‐B) acclimation, herbicide application stops photosynthetic electron transport within 1 h. ROS accumulate, regrowth is inhibited, and plant survival rates are very low. Absorption of low‐dose UV‐B by the UVR8 photoreceptor before herbicide application leads to UVR8 monomerisation and the initiation of signalling pathways promoting ROS defences. REPRESSOR OF UV‐B PHOTOMORPHOGENESIS (RUP) proteins are induced, which then facilitate UVR8 redimerisation, attenuating signalling. Active UVR8 promotes the accumulation of antioxidant defences through multiple mechanisms. Flavonoids are increased through transcriptional upregulation of the CHALCONE SYNTHASE (CHS) enzyme, *CAT3* abundance is increased, carotenoid production is elevated, and increased quenching of excited Chl occurs via nonphotochemical quenching (NPQ). Following UV‐B acclimation, the inhibition of herbicide‐induced electron transport is slowed, facilitating regrowth and increasing plant survival.

Understanding how environmental factors impact herbicide efficacy is essential for effective crop protection management. Alterations in light and temperature can alter the growth, development and photosynthetic activity of plants, affecting herbicide uptake, transport and metabolism (Varanasi *et al*., 2016). In addition, exposure to mild environmental stresses can promote endogenous resilience to higher levels of stress in a process termed priming (Liu *et al*., 2022). In this work, we show that UV‐B acclimation, mediated by the UVR8 photoreceptor, enhances the photosynthetic resilience and survival rate of adult Arabidopsis plants following spraying with multiple widely used herbicides. The effectiveness of UV‐B acclimation appears to depend on herbicide mode of action, with protection focussed on photosystem resilience and antioxidant activity. The absence of protection in *uvr8‐6* mutants and enhanced protection in *rup1/2* mutants supports a role for UVR8 signalling in this response (Figs 4, 5). It is likely that UVR8‐mediated herbicide tolerance operates through multiple signalling pathways to reduce oxidative damage rather than a single mechanism. These include increased energy dissipation via carotenoid production (Fig. 3b) and enhanced NPQ (Figs 3a, 4e) and elevated antioxidant activity (Fig. 5c) induced, in part, from elevated *CAT3* and flavonoid production via *CHS* (Fig. S3). Increasing energy dissipation and reducing oxidative damage postherbicide application is likely to contribute to the delay in ETR (Figs 2, 4, 5), facilitating regrowth from shoot meristems and enhancing the survival rate (summarised in Fig. 6).

The short length of UV‐B acclimation treatment, phenotypic similarity of UV‐B‐acclimated and nonacclimated plants (Figs 1b, 4c, 5b) and reversibility of the protective response in these experiments (Fig. S2) suggest that it is unlikely to result from plant architectural adaptations or UV‐B‐mediated changes in leaf wax synthesis, reducing herbicide diffusion into leaves (Ivaschenko & Ivaschenko, 2019; Zhang *et al*., 2025). Biochemical analyses of leaf waxes would, however, need to be performed to confirm the latter (Wang *et al*., 2007; Yin *et al*., 2012). Similarities in ATZ dose–response relationships between UV‐B‐acclimated and nonacclimated plants suggest that the acclimation treatment is not adversely affecting herbicide uptake or metabolism, but biokinetic analyses would be required to confirm this (Fig. 1a). UV‐B‐induced sunscreen production is thought to be a key adaptation in the evolution of land plants, facilitating terrestrial colonisation in high UV‐B environments (Rozema *et al*., 1997). The UVR8 photoreceptor is highly conserved across the green lineage, with conserved mechanisms of action recorded in green algae, bryophytes and higher plants (Fernández *et al*., 2016; Tilbrook *et al*., 2016; Soriano *et al*., 2018). Accumulating evidence suggests that UV‐B acclimation promotes photosynthetic resilience to abiotic stress through modifying photosystem function in a variety of organisms. In *C. reinhardtii*, UV‐B acclimation enhances NPQ to protect photosystem damage in high light (Tokutsu *et al*., 2021) and maintains the PSII proteins D1 and D2 during UV‐B stress (Tilbrook *et al*., 2016). A role for UV‐B acclimation in the maintenance of D1 protein levels and *F*_v_/*F*_m_ during UV‐B stress has also been observed in Arabidopsis (Leonardelli *et al*., 2024). This response was absent in *uvr8‐6* mutants and enhanced in *rup1/2* mutants, suggesting that UVR8 signalling primes UV‐B and herbicide stress resilience through similar mechanisms.

Collectively, our data suggest that UVR8‐mediated photoprotective mechanisms which reduce sunlight damage to chloroplasts additionally prime plants against the action of multiple widely used herbicides targeting photosystem disruption and oxidative stress. It is likely that UVR8 signalling contributes to discrepancies between herbicide trials in the field and glasshouses, where UV‐B is filtered (Clark *et al*., 2004). Observations that UV‐B filtering during daylight acclimation increases herbicide efficacy in *Chenopodium* suggest that UV‐B‐mediated enhancement of photosynthetic resilience extends beyond Arabidopsis, with potential significance for agricultural weed control. The effect of environmental signalling on plant resilience should therefore be considered alongside effects on product stability, uptake and metabolism when assessing herbicide efficacy. Studies in Arabidopsis have shown that the circadian clock gates glyphosate sensitivity, with maximum effectiveness at dawn (Belbin *et al*., 2019). Optimising the time‐of‐day of application may therefore increase efficacy, reducing application rate. Our data suggest that spraying of herbicides which induce ROS and photosystem damage is likely to be more effective in lower UV‐B environments or following prolonged cloud cover. At smaller scales, UV‐B filtering before herbicide spraying would reverse the protective effects of UV‐B acclimation, enhancing herbicide efficacy. Prolonged UV‐B filtering may, however, cause adverse impacts to crops, such as enhancing shade avoidance, reducing antioxidant capacity and stress resilience, altering flavour/aroma and increasing susceptibility to pests and pathogens (Wargent *et al*., 2011; Hayes *et al*., 2014; Fraser *et al*., 2017; Neugart & Schreiner, 2018). Field studies assessing the impact of UV‐B filtering on crops and weeds in parallel would be required to determine optimum treatment regimes. At larger scales and in cooler geographical locations, the development of nontoxic, spray‐based molecular UV‐B filters could potentially overcome the practical limitations presented by physical shielding, while simultaneously boosting crop yield via leaf heating (Woolley *et al*., 2025). Concerns regarding the impact of herbicides on human health, terrestrial and aquatic ecosystems are increasing (Mohd Ghazi *et al*., 2023). Greater understanding of how environmental signalling pathways are integrated with the circadian clock to shape plant herbicide resilience is central to optimising herbicide design and application, reducing dosage and minimising risks to both workers and the environment.

## Competing interests

None declared.

## Author contributions

CLG and KAF planned and designed the research. CLG performed the experiments. CLG and KAF analysed data and wrote the manuscript.

## Disclaimer

The New Phytologist Foundation remains neutral with regard to jurisdictional claims in maps and in any institutional affiliations.

## Supporting information


**Fig. S1** Spectral photon irradiance of plant growth conditions.
**Fig. S2** ATZ protection provided by UV‐B acclimation is transient.
**Fig. S3** UVR8 signalling enhances *CHS* and *CAT3* transcript accumulation.
**Fig. S4** UVR8 signalling enhances tolerance to multiple herbicides.
**Fig. S5** UV‐B attenuation during daylight acclimation increases the efficacy of the herbicide Calaris on *Chenopodium amaranticolor*.
**Table S1** Primer sequences used in this study.
Please note: Wiley is not responsible for the content or functionality of any Supporting Information supplied by the authors. Any queries (other than missing material) should be directed to the *New Phytologist* Central Office.

## Data Availability

The data that support the findings of this study are available in the article and in the Supporting Information (Figs S1–S5; Table S1).
