# Supplementary material for: UV RESISTANCE LOCUS 8 signalling enhances photosynthetic resilience to herbicide‐induced damage in Arabidopsis thaliana
Source: New Phytol. 2025 Jun 14;247(4):1763–76. doi: 10.1111/nph.70303 (PMC12267928; doi:10.1111/nph.70303)
Supplement: Supplementary file 1 — Fig. S1 Spectral photon irradiance of plant growth conditions. Fig. S2 ATZ protection provided by UV‐B acclimation is transient. Fig. S3 UVR8 signalling enhances CHS and CAT3 transcript accumulation. Fig. S4 UVR8 signalling enhances tolerance to multiple herbicides. Fig. S5 UV‐B attenuation during daylight acclimation increases the efficacy of the herbicide Calaris on Chenopodium amaranticolor. Table S1 Primer sequences used in this study. Please note: Wiley is not responsible for the content or functionality of any Supporting Information supplied by the authors. Any queries (other than missing material) should be directed to the New Phytologist Central Office. [file NPH-247-1763-s001.pdf]

***New Phytologist* Supporting Information**

Article title: UV RESISTANCE LOCUS 8 (UVR8) signalling enhances photosynthetic resilience to herbicide-induced damage in *Arabidopsis thaliana*

Authors: Christopher L. Groves and Keara A. Franklin

Article acceptance date: 27 May 2025

The following Supporting Information is available for this article:

Fig. S1. Spectral photon irradiance of plant growth conditions.
Fig. S2. ATZ protection provided by UV-B acclimation is transient.
Fig. S3. UVR8 signalling enhances *CHS* and *CAT3* transcript accumulation.
Fig. S4. UVR8 signalling enhances tolerance to multiple herbicides.
Fig. S5. UV-B attenuation during daylight acclimation increases the efficacy of the herbicide Calaris on *Chenopodium amaranticolor*.
Table S1. Primer sequences used in this study.

Fig. S1. Spectral photon irradiance of plant growth conditions.

(a) Spectral comparison of white light (PAR) and white light supplemented with 1 µmolm^{-2}s^{-1} narrowband UV-B at peak 311 nm (PAR + UV-B). (b) Spectral comparison of daylight in Bristol, UK (August 2023), with and without a UV-B-blocking polycarbonate filter.

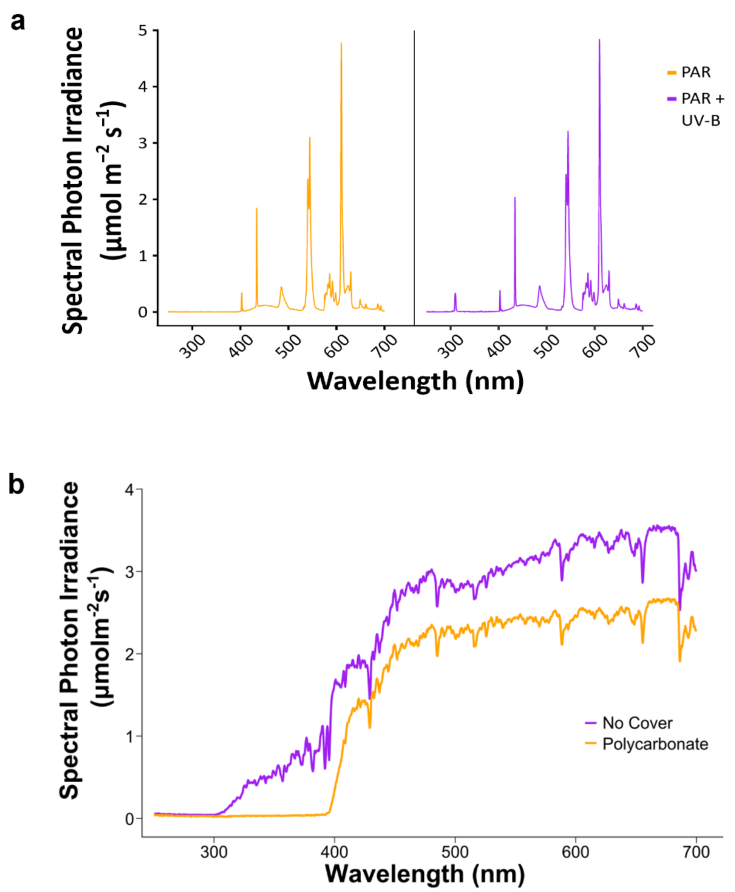

Figure S2: **ATZ protection provided by UV-B acclimation is transient.**

F_v/F_m of Arabidopsis plants subject to UV-B acclimation at different times prior to ATZ spraying. Plants were grown on soil in white light (PAR: 80 µmol m^{-2} s^{-1}) at 20°C, prior to ATZ spraying on day 24. ATZ was sprayed at 20 gai/ha in a 10% acetone + 0.1% Genapol X-080 formulation at 2 h post dawn and Fv/Fm recorded 24 h later. To assess the longevity of UV-B acclimation, groups of plants were treated with 2 days of supplementary narrowband UV-B (1 µmol m^{-2} s^{-1}) at 0, 2, 4 or 7 days prior to ATZ spraying. A one-way ANOVA and Tukey's post-hoc test were used to identify differences between treatments. Data are presented as boxplots showing the median and interquartile range of each group. The upper and lower whiskers represent data within 1.5 * IQR. Different letters represent significant differences ($P < 0.05$). n = 8.

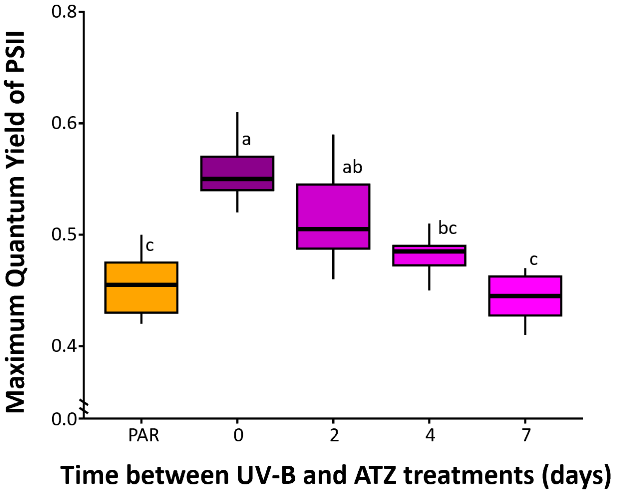

Figure S3. UVR8 signalling enhances *CHS* and *CAT3* transcript accumulation.

The effect of UV-B acclimation on relative transcript abundance of *CHS, PsbA, GSTF8*, *APX6* and *CAT3* in Col-0, *uvr8-6* and *rup1/2*, before and after ATZ treatment. Arabidopsis plants were grown on soil for 21 d in white light (PAR: 80 µmol m^{-2} s^{-1}) at 20°C. Half the plants were then treated with supplementary narrowband UV-B at 1 µmol m^{-2} s^{-1} for 2 d prior to ATZ treatment (PAR+ UV-B). ATZ was sprayed at 80 gai/ha in a 10% acetone + 0.1% Genapol X-080 formulation at 2 h post dawn. Rosette leaf 2 was harvested at 0, 1, 2 and 4 h post spray. Relative transcript abundance was calculated using *ACT2* as a reference. A two-way ANOVA and Tukey's post-hoc test was used to identify differences between treatments at each timepoint. n=3. Bars represent mean transcript abundance, and error bars represent SEM.

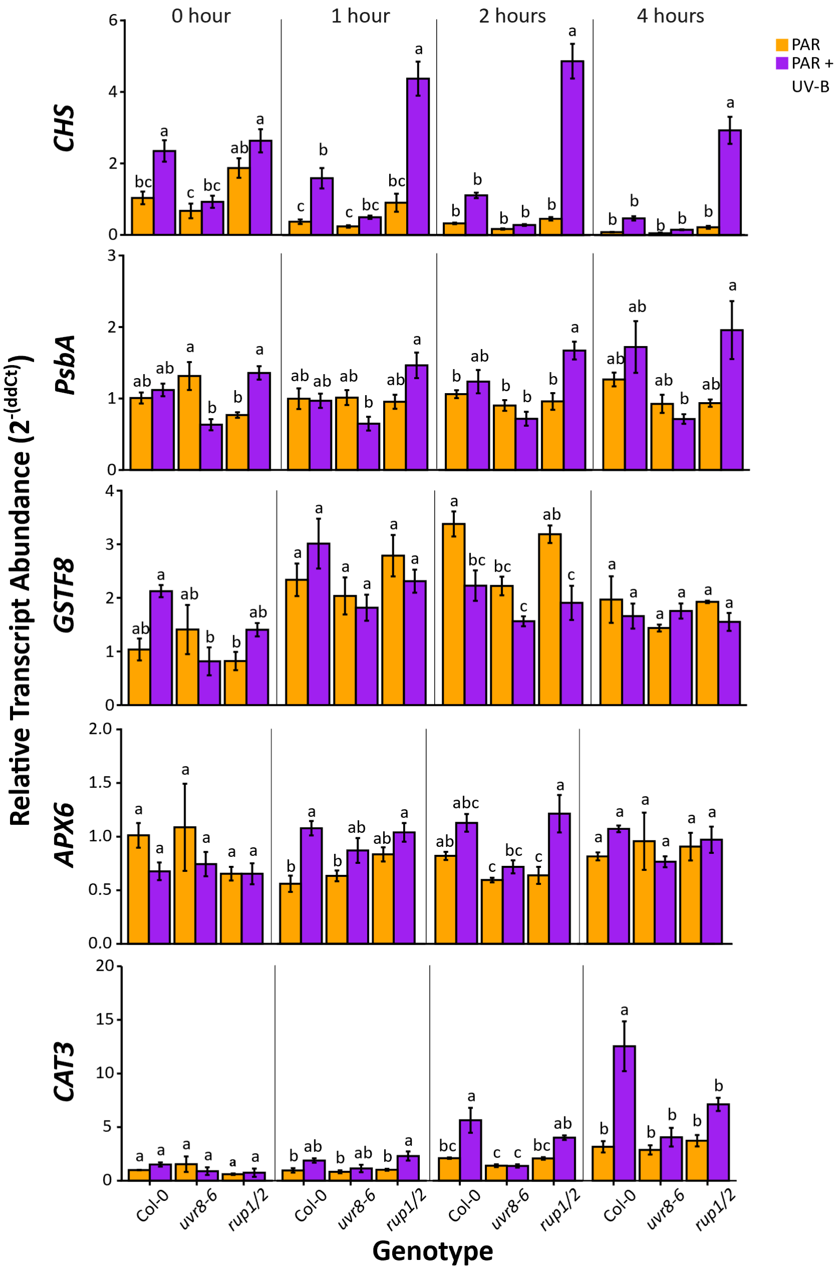

Figure S4. UVR8 signalling enhances tolerance to multiple herbicides.

F_v/F_m of Arabidopsis Col-0 and *uvr8-6* plants following (a) Mesotrione, (b) Metribuzin (c) Glyphosate, (d) Roundup NL Glyphosate Free (Pelargonic Acid) and (e) Weedol (Fluroxypyr, Clopyralid and MCPA (2-methyl-4-chlorophenoxyacetic acid)) treatment. Plants were grown on soil for 21 d in white light (PAR: 80 µmol m^{-2} s^{-1}) at 20°C. Half the plants were then treated with supplementary narrowband UV-B at 1 µmol m^{-2} s^{-1} for 2 d prior to herbicide treatment (PAR+ UV-B). All herbicides were sprayed at 2 h post dawn and F_v/F_m measurements recorded 24, 48, 72 and 120 h later. Mesotrione was dissolved in water and sprayed at 0.625 and 1.25 gai/ha (a). Metribuzin was dissolved in 0.1% Genapol X-080 at 5 and 10 gai/ha (b). Glyphosate was dissolved in water and applied at 12.5 and 25 gai/ha (c). Roundup NL Glyphosate Free (d) and Weedol (e) were bought as prepared products and applied at 1x and 0.5x the product strength. Dilution was prepared with RO water. A two-way ANOVA and Tukey's post-hoc test were used to identify differences between treatments. Data are presented as boxplots showing the median and interquartile range of each group. The upper and lower whiskers represent data within 1.5 * IQR. Dots represent outliers. Different letters represent significant differences (P < 0.05). n = 6-8.

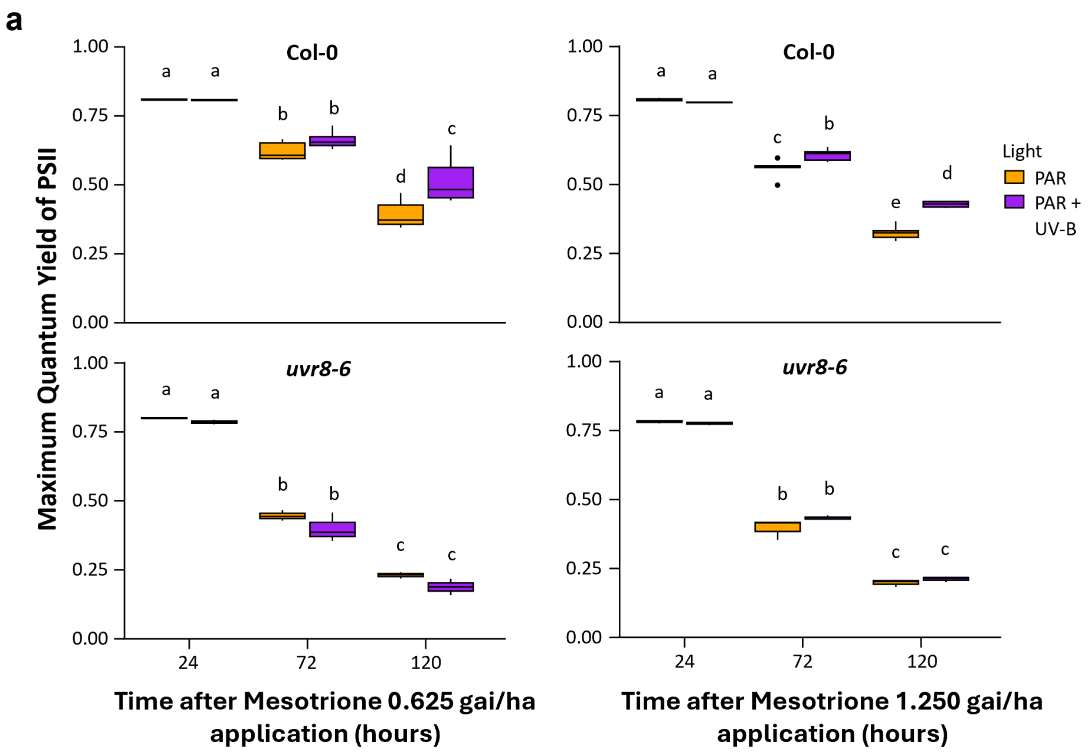

b

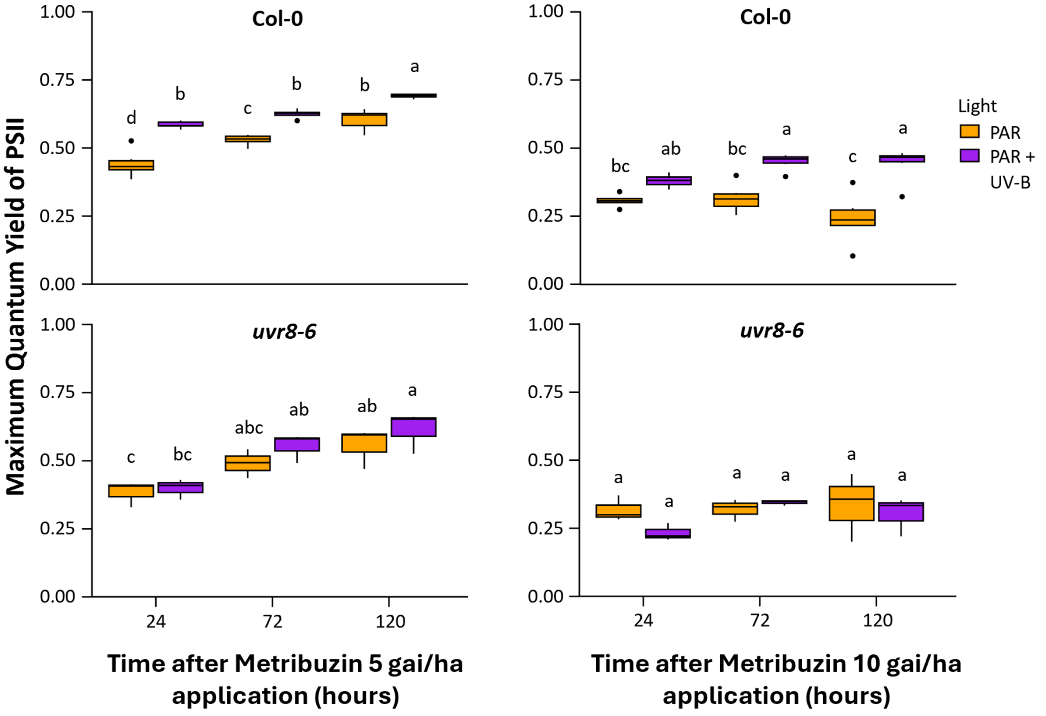

Col-0
uvr8-6
Maximum Quantum Yield of PSII
1.00
0.75
0.50
0.25
0.00
24
72
120
Time after Metribuzin 5 gai/ha application (hours)
Time after Metribuzin 10 gai/ha application (hours)
Light
PAR
PAR + UV-B

c

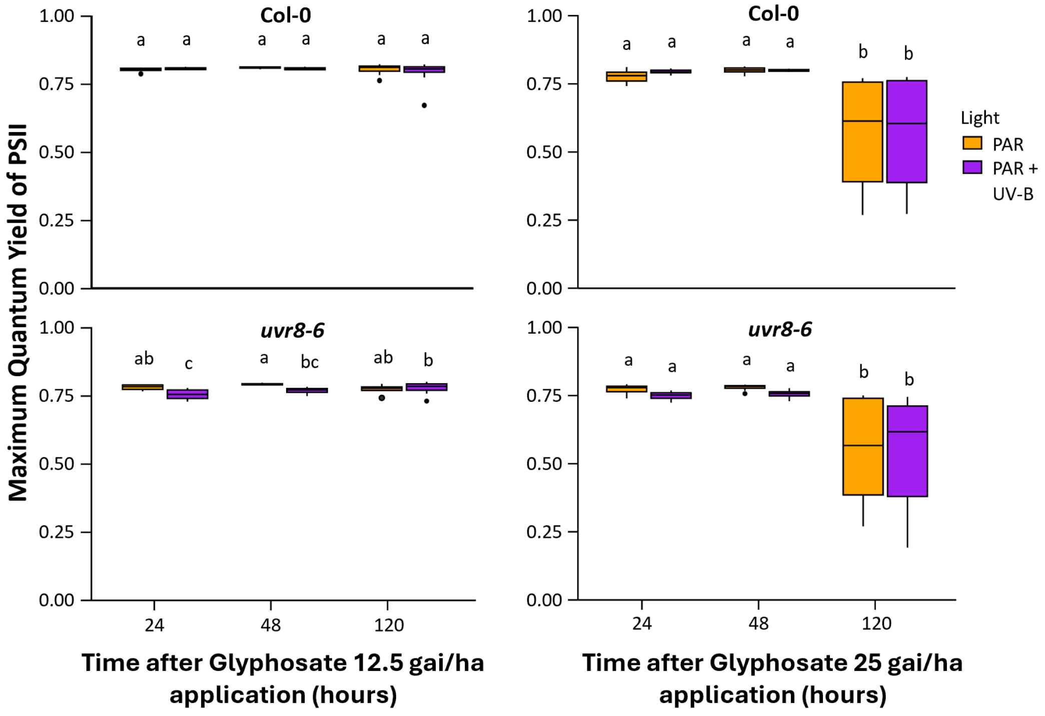

Col-0
uvr8-6
Maximum Quantum Yield of PSII
1.00
0.75
0.50
0.25
0.00
24
48
120
Time after Glyphosate 12.5 gai/ha application (hours)
Time after Glyphosate 25 gai/ha application (hours)
Light
PAR
PAR + UV-B

d

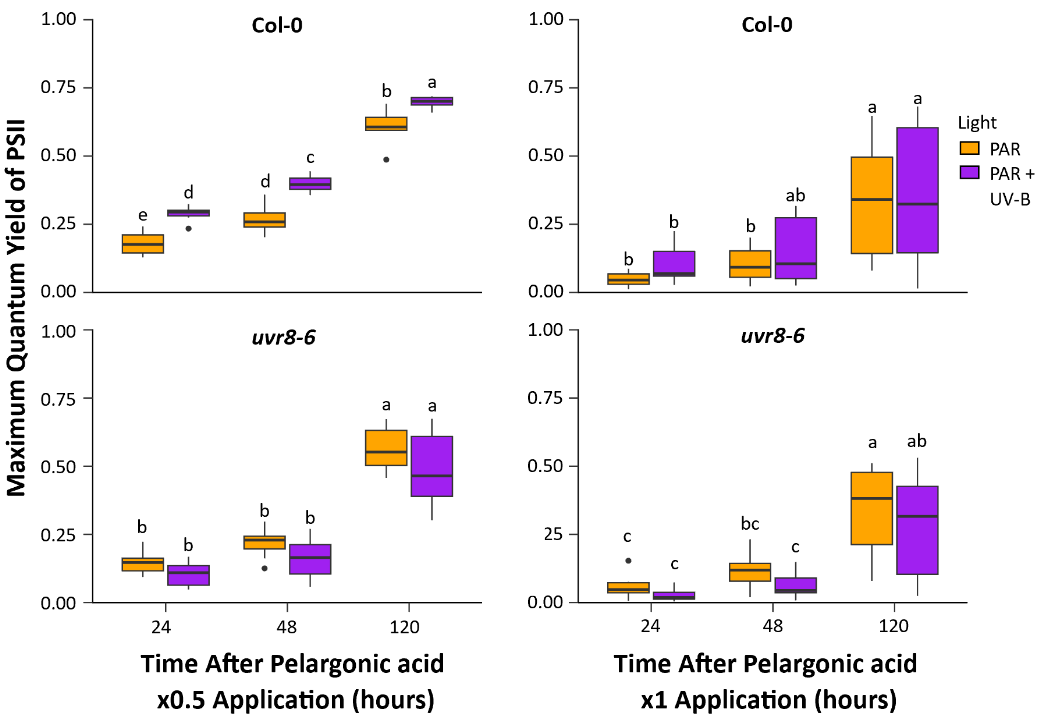
Col-0
Col-0
uvr8-6
uvr8-6
Maximum Quantum Yield of PSII
1.00
0.75
0.50
0.25
0.00
Light
PAR
PAR + UV-B
24
48
120
Time After Pelargonic acid x0.5 Application (hours)
Time After Pelargonic acid x1 Application (hours)

e

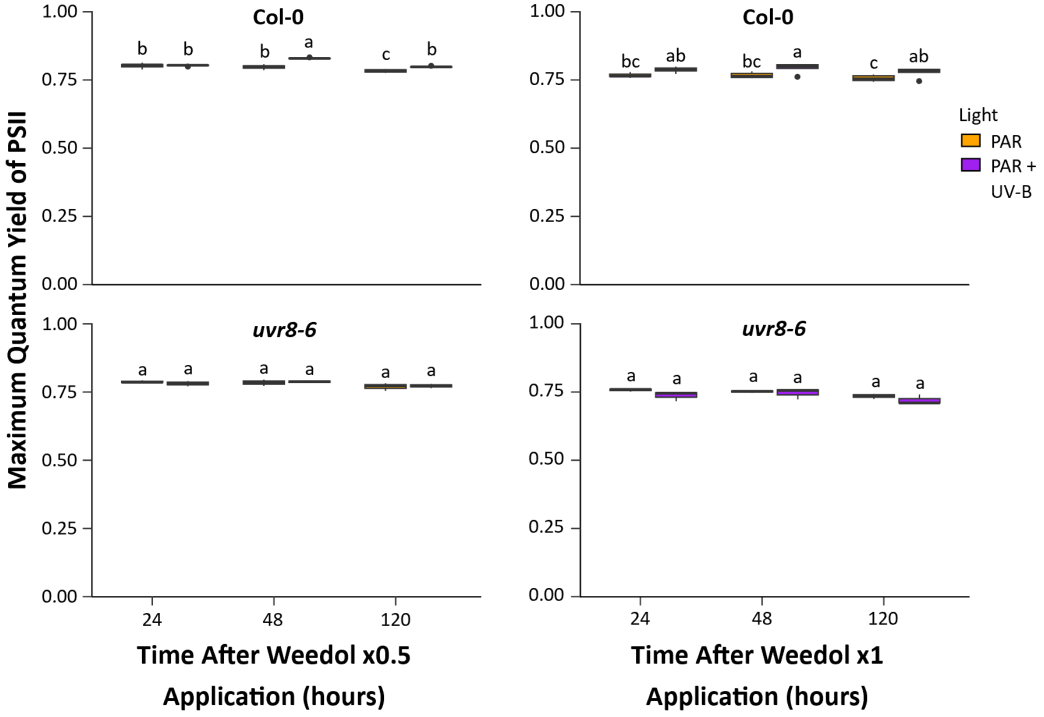
Col-0
Col-0
uvr8-6
uvr8-6
Maximum Quantum Yield of PSII
1.00
0.75
0.50
0.25
0.00
Light
PAR
PAR + UV-B
24
48
120
Time After Weedol x0.5 Application (hours)
Time After Weedol x1 Application (hours)

Figure S5: **UV-B attenuation during daylight acclimation increases the efficacy of the herbicide Calaris on *Chenopodium amaranticolor*.**

Chenopodium plants were grown in a glasshouse for 14 days at 22°C, then moved outside in Bristol, UK (August 2023) for 3 days. Weather conditions were cloudy but dry. Plants were acclimated in daylight (No Cover; UV-B at ~ 1 µmol$m^{-2}s^{-1}$) or covered by a UV-B filtering polycarbonate sheet positioned overhead. Plants were then sprayed with 4 rates of Calaris at 12.5, 25, 50 and 100 gai/ha and returned to the glasshouse. (a) Fv/Fm analyses of plants following herbicide spraying. A two-way ANOVA was used to determine differences between light treatments at each time point for each rate, with significant differences shown as p<0.5 *, p<0.01 ** and p<0.001 ***. (b) Photographs of plants 14 days after spraying and % survival rates, determined by the presence of an active shoot apical meristem and newly produced green tissue. n = 8.

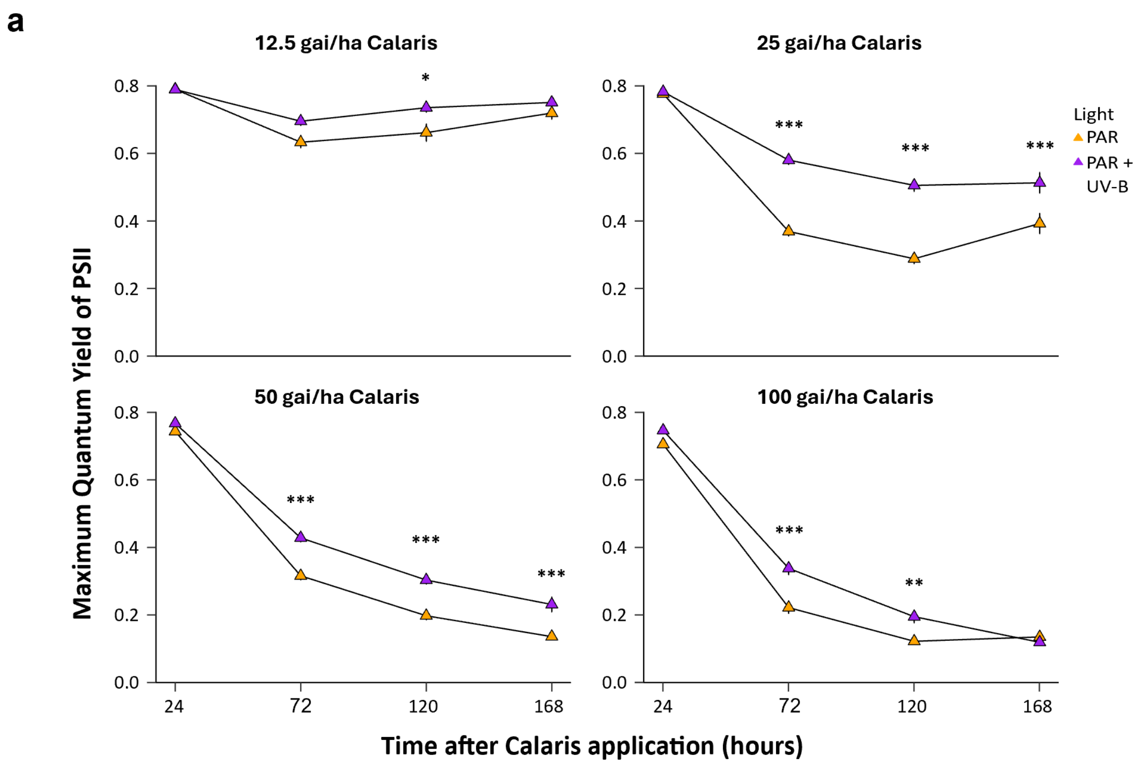

b

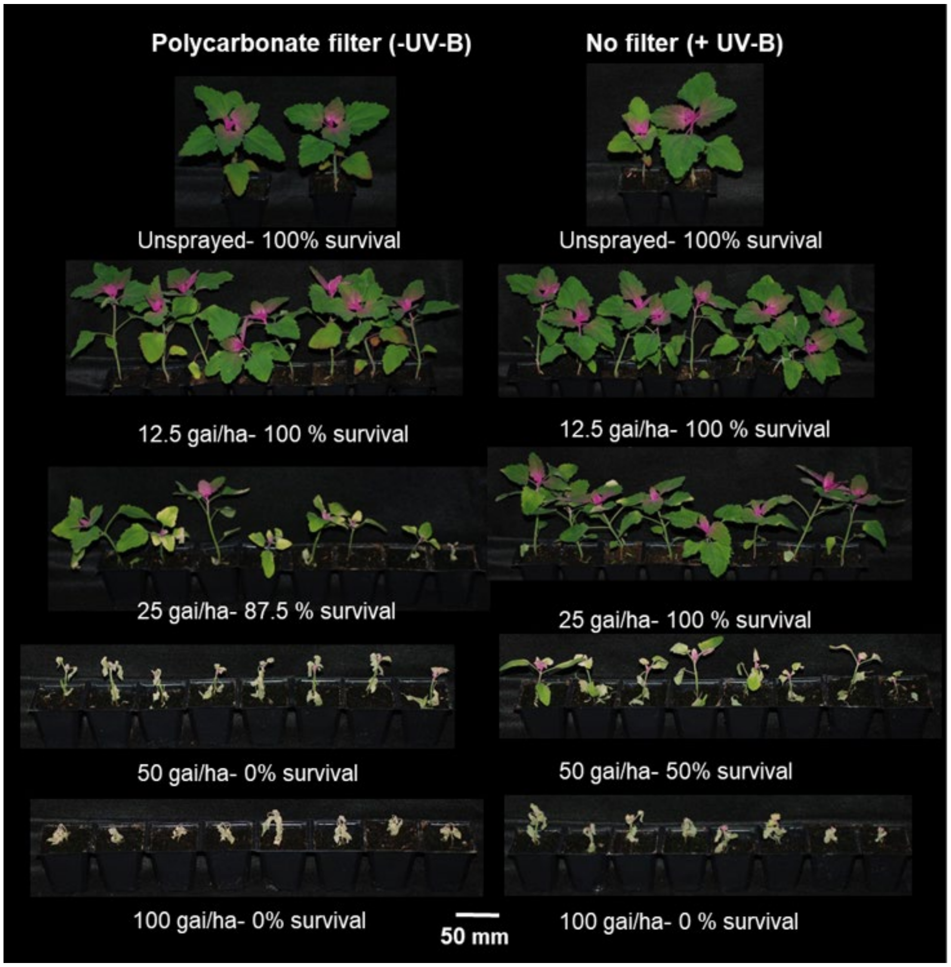

Table S1. Primer sequences used in this study.

Gene	Sequence
ACT2	FW: 5’- TCAGATGCCCAGAAGTGTTGTTCC- 3’
	RV: 5’ -CCGTACAGATCCTTCCTGATATCC- 3’
CHS	FW: 5´-TTCCGCATCACCAACAGTGAAC-3´
	RV: 5´-CGCACATGCGCTTGAACTTCTC-3´
PsbA	FW: 5´-ATACAACGGCGGTCCTTATGAAC-3´
	RV: 5´-CAAGGACGCATACCCAGACGG-3´
GSTF8	FW: 5´-ACCTTGCCCTCAACCCCTT-3´
	RV: 5´-AGGTACTGTGTGATGGCTCTTG-3´
APX6	FW: 5´-CTCCAGAAACCCTGAGTGCC-3´
	RV: 5´-AGCTCCTGGGTCGAAAATCC-3´
CAT3	FW: 5´-TCAAGTTTTACACCAGAGAGGGAA-3´
	RV: 5´-AACATCCGGGAACTGAATCCC-3´
